# Acceptability and effectiveness of opportunistic referral of smokers to telephone cessation advice from a nurse: a randomised trial in Australian general practice

**DOI:** 10.1186/1471-2296-9-16

**Published:** 2008-02-28

**Authors:** Jane M Young, Seham Girgis, Tracey A Bruce, Melissa Hobbs, Jeanette E Ward

**Affiliations:** 1 Surgical Outcomes Research Centre, Sydney South West Area Health Service, Australia; 2 School of Public Health, University of Sydney, Australia; 3 The Diabetes Unit, Australian Health Policy Institute, The University of Sydney, Australia; 4 Injury and Musculoskeletal Division, The George Institute for International Health, Australia; 5 Policy Implementation in Population Health, Canada Research Chair, Australia

## Abstract

**Background:**

GPs often lack time to provide intensive cessation advice for patients who smoke. This study aimed to determine the effectiveness of opportunistic referral of smokers by their GP for telephone cessation counselling by a trained nurse.

**Methods:**

Adult smokers (n = 318) attending 30 GPs in South Western Sydney, Australia were randomly allocated to usual care or referral to a telephone-based program comprising assessment and stage-based behavioural advice, written information and follow-up delivered by a nurse. Self-reported point prevalence abstinence at six and 12 months was compared between groups. Characteristics of patients who accepted and completed the intervention were investigated.

**Results:**

Of 169 smokers randomised to the intervention, 76 (45%) consented to referral. Compared with smokers in 'pre-contemplation', those further along the stage-of-change continuum were significantly more likely to consent (p = 0.003). Those further along the continuum also were significantly more likely to complete all four calls of the intervention (OR 2.6, 95% CI: 0.8–8.1 and OR 8.6, 95% CI: 1.7–44.4 for 'contemplation' and 'preparation' respectively). At six months, there was no significant difference between groups in point prevalence abstinence (intention to treat) (9% versus 8%, p = 0.7). There was no evidence of differential intervention effectiveness by baseline stage-of-change (p = 0.6) or patient sex (p = 0.5). At 12 months, point prevalence abstinence in the intervention and control groups was 8% and 6% respectively (p = 0.6).

**Conclusion:**

Acceptance of opportunistic referral for nurse delivered telephone cessation advice was low. This trial did not demonstrate improved quit rates following the intervention. Future research efforts might better focus support for those patients who are motivated to quit.

**Australian Clinical Trials Registry number:**

ACTRN012607000091404

## Background

Australian general practice provides a unique setting for the provision of smoking cessation advice as over 80% of the population visits a general practitioner (GP) in any given year [1]. An 'opportunistic' approach to cessation advice in which smoking is discussed with every patient, irrespective of the reason for the consultation, has the potential to reach a large number of smokers in the community [2]. Despite compelling evidence for their impact [3-5] and their unrivalled credibility as providers of health-related information [6], Australian GPs persistently exhibit low rates of detection of smokers and provision of evidence-based smoking cessation advice [7-10]. Those few randomised trials conducted in Australia to redress this gap between evidence and practice provide no clear-cut conclusions that any strategy will work to increase GPs' systematic identification of smokers or enhance rates of advice [11-14].

GPs themselves perceive lack of time to be one of the major impediments to their intervention with smokers [7,10]. Smoking cessation advice, particularly those intensive and time-consuming interventions known to be especially effective in promoting quit attempts and achieving abstinence, could perhaps be delegated to and delivered by a nurse [15,16]. A recent meta-analysis of 30 trials of nurse-delivered cessation advice found a modest overall positive effect when compared with controls or usual care (OR 1.47, 95%CI: 1.29–1.68) [17]. A subgroup analysis of those 11 studies involving smokers in primary care or out-patient settings indicated that smoking intervention by nurses almost doubled the odds of success (OR 1.90, 95%CI 1.48–2.43) [17]. In all of these studies, smokers were required to attend at least one face-to-face session, either individually or as part of a group. While a component of some interventions, telephone counselling was used only as an adjunct to face-to-face counselling and was not offered as a convenient medium for advice.

Cessation counselling that is delivered entirely by telephone holds considerable promise as a relatively inexpensive means to deliver individual advice that also can be flexible and tailored to individual needs. Telephone counselling has been shown to increase quit rates significantly compared with minimal or usual care when provided 'reactively' in response to a smoker's contact with a quit-line or as an adjunct to hospital-initiated programs [18,19] and has been recommended by several national guidelines for tobacco control [4,20-22].

A recent Cochrane Review of 29 controlled trials of 'proactive' telephone counselling reported only a modest overall benefit however [23]. A subgroup analysis of 19 studies found that proactive telephone cessation advice had minimal impact when provided to smokers who were recruited irrespective of their current intention to quit [23]. Only three studies in this group involved recruitment of patients through primary care. The only study that recruited smokers opportunistically did not find any additional benefit from telephone follow up used to reinforce physician advice, regardless of the intensity of that advice [24].

Thus, there remains a paucity of rigorous scientific evidence for or against opportunistic referral of smokers for telephone-delivered smoking cessation advice. As practice nurses are a rarity in Australian general practice, assessment of the acceptability to patients of referral to a nurse for health promotion advice is particularly important. In response, we designed this randomised trial to determine acceptability and the effectiveness of this approach to improve quit rates and shifts in stage-of-change in smokers after six and twelve months. We also investigated the characteristics of smokers who consented to and completed this intervention.

## Methods

The study was approved by the Ethics Review Committees (RPAH and Liverpool zones) of Sydney South West Area Health Service (Protocol number X01-0034).

### GP recruitment

From a list of all GPs in South Western Sydney (SWS)(n = 752), we selected 165 GPs who practised in two postcode zones with the highest proportion of English-speaking residents [25] and accessible to the research team. GPs were eligible if they worked at least 24 hours per week in this practice; estimated that at least 60% of their patients spoke English and were in practice during the patient recruitment period. We excluded GPs working in medical centres with more than five GPs as continuity of care is not a feature of these centres in the Australian health care system. During October 2003 – July 2004, eligible GPs were approached and recruited for the study in waves, using a step-wise approach used previously to achieve high participation rates [26]. Reception staff were briefed in the study's requirements.

### Patient recruitment

During a three week study period at each practice, all patients aged 18–65 years attending for routine consultations were approached in the waiting room and were given an information letter and self-administered questionnaire to complete before seeing their GP. Patients were considered ineligible if they were unable to read or understand the information sheet, did not speak sufficient English, were in distress, were planning to leave Australia during the study period or had previously been approached. Patients who did not wish to participate were asked to provide their age and sex to enable us to monitor possible response bias.

The pre-consultation questionnaire ascertained routine demographic data and self-reported smoking status. Previous Australian research has confirmed the accuracy of self-report to be high with sensitivity and specificity of 94% [27]. Overseas studies also suggest that the validity of self-reported smoking is high, discouraging the need for cotinine validation [28,29]. Patients who self-reported they were smokers were further asked about nicotine dependence [30]; stage-of-change ('Pre-contemplation', 'Contemplation', 'Preparation') [31] and previous quit attempts. All patients were instructed to hand their completed questionnaire to their GP at the start of the consultation.

### Randomisation and study plan

We had pre-randomised questionnaires and allocated unobtrusive marks that were meaningful only to the GPs in order to convey group allocation. Questionnaires were randomly ordered and coded prior to delivery to the practice by selecting sequential numbers from a computer generated random number list [32]. Patients were thereby assigned either to intervention or control groups in the waiting room before they consulted their GP.

GPs scanned questionnaires during the consultation to determine smoking status and group allocation. Control group smokers received the GP's usual care. We also provided GPs with free copies of government-sponsored quit kits [33] to distribute to smokers in this group. Intervention group smokers received an offer from their GP of free referral for telephone-based cessation advice from a trained nurse. Intervention group patients who declined referral were included in our intention-to-treat analyses but not contacted by the nurse. Practices were visited by research staff twice each week during the study period to collect completed questionnaires and study logs and to liaise with practice staff.

### Intervention details

Within three days, a nurse trained in smoking cessation [34] telephoned each patient. Our telephone-delivered intervention, based on the '5As' approach [4,35], was structured using an explicit protocol to ensure consistency. During the first call, smoking history, nicotine dependence and previous quit attempts were assessed. Smokers then were asked if they were willing to make a quit attempt. Those who were not ready to do so received a motivational intervention based on the '5Rs' to assist their progression along the 'stage-of-change' continuum [4]. Smokers who were ready to make a quit attempt were assisted to set a specific quit date and to plan their quit attempt. The nurse also mailed them a quit kit [33]. Unless contra-indicated, these smokers were encouraged to purchase nicotine replacement therapy (available in Australia without a doctor's prescription) to manage nicotine withdrawal. Smokers who set a quit date were telephoned again on the specified quit day, then one week and three weeks after the quit date. During these three calls, participants were congratulated if they had quit, were encouraged to maintain quitting and assisted in resolving any problems arising. Relapsing smokers received motivation advice and were encouraged to 're-frame' relapse as a learning experience for future cessation.

### Behavioural and psychological outcome measures

Follow-up questionnaires were mailed at six and twelve months. A standardised follow-up protocol was followed to maximise return rates. After 1 month, remaining non-respondents were asked to complete a short telephone interview with a research assistant who was blind to their group allocation to obtain a minimum data-set for study outcomes.

### Sample size

Based on previous research demonstrating that differences in quit rates of over 10% can be achieved with intensive smoking cessation interventions involving individualised behavioural counselling and repeated follow up [4] and assuming that baseline smoking prevalence would be 20%, we calculated that 214 smokers would be needed in each group in order to detect a 10% difference in quit rates at 12 months, with 80% power and 5% alpha. A pilot study conducted in three general practices found very high acceptance of the intervention among smokers with only 5% declining telephone assistance from a nurse [36].

### Statistical analyses

Smoking status and stage-of-change for participants who were lost to follow-up were considered to be unchanged from baseline for the purpose of analysis. Point prevalence abstinence at six and twelve months was compared between groups on an intention to treat basis using chi-squared tests. To investigate the effect of patient sex and baseline stage of change on intervention effectiveness, subgroup analyses were performed. Interaction terms between sex and group and stage-of-change and group were created and entered into logistic regression models. The significance of each interaction term was assessed to determine whether the intervention effect differed among the subgroups [37].

The proportion of participants who had made at least one quit attempt lasting at least 24 hours and mean shift in stage-of-change between baseline and 6 months and baseline and 12 months were compared between groups using chi-square tests and t tests respectively [38].

Characteristics of patients in the intervention group who were willing to be contacted by the nurse counsellor were compared with those who declined. Logistic regression modelling was then used to identify significant, independent predictors of those who agreed to the intervention. Similarly, predictors of smokers who completed all of the counselling sessions were investigated. All analyses were undertaken using SAS statistical software [32].

## Results

### GP recruitment

Of 67 eligible GPs in the selected two postcodes, 35 (52%) agreed to participate but five withdrew before recruiting patients. Twenty-four (80%) participating GPs were male and six (20%) were female; 15 (50%) were in solo practices, 29 (97%) worked full-time. There were no significant differences between eligible GPs who participated compared with those who did not in regard to gender (p = 1.0); working hours (p = 1.0) and working in solo or group practice, (p = 0.8).

### Patient recruitment

Of 1888 patients who were approached to participate, 472 (25%) were ineligible: poor English (n = 150); unable to read (n = 79); cognitive impairment (n = 88); too sick (n = 58); already been approached (n = 86); about to go overseas (n = 2); acutely unwell (n = 9). Of 1416 eligible patients, 1170 (83%) agreed to complete pre-consultation questionnaires. There was no significant difference in the mean age (41.9 versus 41.5 years, p = 0.7) or gender (p = 0.09) of those who did or did not participate. Differences in smoking status could not be assessed as this information was unavailable for half (50%) of those who declined participation.

### Baseline characteristics of participants

**Figure 1:**
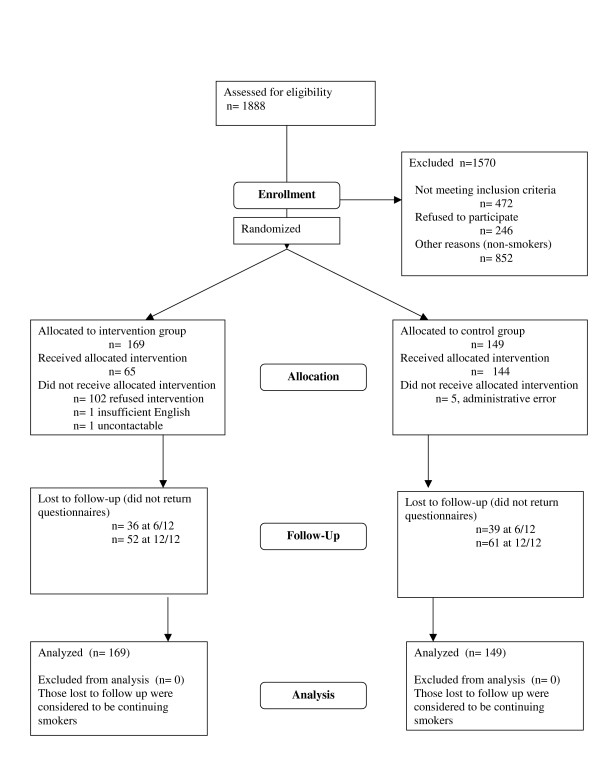
Randomised trial of opportunistic referral of smokers by GPs to telephone cessation advice from a nurse: CONSORT flowchart.

Of the 1170 participating patients, 318 (27%) were smokers (Figure 1). Of these, 169 were randomly allocated to the intervention group and 149 to the control group. Characteristics of participants in each group are summarised in Table 1.

**Table 1:** Baseline characteristics of smokers assigned to intervention and control groups

**Characteristics**	**Intervention group (N = 169) n (%)**	**Control group (N = 149) n (%)**
***Demographic characteristics***		
Age (mean ± SD years)	39 (12)	36 (12)
Male	87 (51)	61 (41)
Completed high school or tertiary education	70 (41)	59 (40)
Employed full or part-time	102 (60)	88 (59)
English language spoken at home	153 (91)	139 (93)

***General health characteristics***		

'Excellent' or 'Very good' self-reported health status	42 (25)	40 (27)
Concurrent conditions (self-reported)		
Heart disease	5 (3)	4 (3)
Chronic bronchitis or emphysema	5 (3)	8 (5)
Cancer	1 (<1)	2 (2)
Diabetes mellitus	7 (4)	8 (5)
Vascular disease	7 (4)	5 (3)
Attending regular GP	134 (79)	120 (80)

***Smoking characteristics***		

Smoking status		
Regular smoker	145 (86)	130 (87)
Occasional smoker (not every day)	23 (14)	19 (13)
Pipes and cigars only	1 (<1)	0
Amount smoked (cigarettes/day)		
10 or less	39 (24)	46 (32)
11–20	74 (46)	63 (43)
21–30	38 (24)	28 (19)
31 or more	10 (6)	8 (6)
Nicotine dependency rated 'High' or 'Very High'	50 (30)	40 (27)
Stage of change for smoking cessation		
Pre-contemplation	58 (34)	61 (41)
Contemplation	62 (37)	48 (32)
Preparation	33 (20)	35 (23)
Missing	16 (9)	5 (3)
At least one quit attempt in previous 6 months	64 (38)	36 (24)
At least one quit attempt in previous 12 months	79 (47)	51 (34)

Patient flow through the study is summarised in Figure 1. Five control group patients received the nursing intervention by mistake due to an administrative error. These patients were analysed as controls according to intention to treat principles.

### Patient acceptance of the intervention

Of the 169 smokers allocated to the intervention group, less than half (n = 76, 45%) gave consent to their GP to be referred to a nurse. Smokers who consented were somewhat older than those who declined (mean 38 years versus 35 years, p = 0.05) and were significantly further along the stage-of-change continuum (p = 0.003). There were no other significant associations between characteristics of participants and consent to the intervention (Table 2).

**Table 2:** Characteristics of intervention group participants by willingness to be contacted by the nurse counsellor

**Characteristics**	**Consented (N = 76) n (%)**	**Declined (N = 93) n (%)**	**p-value**
Mean age (years)	38	35	**0.05**
Male	38 (50)	49 (53)	0.7
Completed high school or tertiary education	34 (45)	36 (39)	0.4
Employed full or part-time	46 (61)	56 (60)	1.0
English language spoken at home	70 (92)	83 (89)	0.5
'Excellent' or 'Very good' self-reported health status	17 (22)	25 (27)	0.5
Attending regular GP	64 (84)	70 (75)	0.2
Regular smoker (smoked every day)	68 (89)	77 (83)	0.2
Stage of change for smoking cessation			
*Pre-contemplation*	19 (28)	39 (46)	**0.003**
*Contemplation*	28 (41)	34 (40)	
*Preparation*	22 (32)	11 (13)	
At least one quit attempt in previous 6 months	33 (43)	31 (33)	0.2
At least one quit attempt in previous 12 months	41 (54)	38 (41)	0.09

Of those 76 smokers who initially accepted nurse-delivered counselling, one was subsequently considered ineligible by the nurse in the first telephone contact due to poor English. One smoker could not be contacted and nine refused to proceed with the initial call. Of the remaining 65 smokers, 4 subsequently declined any further support at the conclusion of their initial telephone call. Sixty-one smokers completed the second call and 58 completed the third. Fifty-five smokers completed all four calls of the intervention, representing 33% of those randomised but 72% of those who agreed to and were eligible for, telephone counselling. Among those who initially agreed to receive the intervention, the only significant predictor of completion of all four calls was baseline stage-of-change. Smokers who, at baseline, were in 'contemplation' had a three-fold increase in the odds (OR 2.6, 95% CI: 0.8–8.1) and those in 'preparation' had a nine-fold increase in the odds (OR 8.6, 95% CI: 1.7–44.4) of completing all four calls of the intervention compared with those in 'pre-contemplation'.

### Patient follow-up

Follow-up data were available at six months for 133 (79%) smokers in the intervention group and 110 (74%) in the control group (p = 0.3). At 12 months, response fractions were 69% (117/169) and 59% (88/149) respectively (p = 0.06).

### Outcomes assessment

There were no significant differences in study outcomes between groups at either six or 12 months (Table 3). Logistic regression modelling demonstrated that the intervention was equally ineffective for men and women as the interaction term for sex by group was not significant (Table 4). Likewise, the intervention was equally ineffective for smokers who were in 'pre-contemplation', 'contemplation' or 'preparation' stages of change for smoking cessation at the time of recruitment to the study (Table 4).

**Table 3:** Behavioural and psychological outcomes at six and twelve months

**Outcome**	**Intervention group (N = 169) n %**	**Control group (N = 149)**	**Relative Risk (95% confidence interval)**
**At six months**			
Quit smoking	15 (9)	12 (8)	1.1 (0.5–2.3)
Quit at least once for more than 24 hours in past 6 months	54 (32)	44 (30)	1.1 (0.8–1.5)
Mean number of stage shifts in stage of change from baseline	0.01	0.0	Mean difference = 0.01 (-0.1 – 0.1)
			
**At twelve months**			
Quit smoking	13 (8)	9 (6)	1.3 (0.6–2.9)
Quit at least once for more than 24 hours in past 12 months	32 (19)	17 (11)	1.7 (0.96–2.9)
Mean number of stage shifts in stage of change from baseline	0.15	0.09	Mean difference = 0.06 (-0.1 – 0.2)

*not evaluable for patients with missing data for baseline stage of change

**Table 4:** Quit rates at 6 months among subgroups

	**Intervention group n/N (%)**	**Control group n/N (%)**	Significance of interaction term*
Sex			
*Men*	8/87 (9)	3/61 (5)	p = 0.5
*Women*	7/82 (9)	9/88 (10)	
			
Stage-of-change			
*'pre-contemplation'*	1/58 (2)	2/61 (3)	p = 0.6
*'contemplation'*	2/62 (3)	2/48 (4)	
*'preparation'*	5/33 (15)	6/35 (17)	

* intervention group (intervention or control) X level of subgroup

### Outcomes in smokers who completed the intervention

Among the 55 smokers in the intervention group who completed all four calls of the intervention, point prevalence abstinence rates at six and 12 months were 16% (9/55) and 13% (7/55) respectively. At six months, 27 (49%) of these smokers had made at least one quit attempt lasting 24 hours or more but the mean stage shift from baseline stage of change was zero. At 12 months, only 18 (33%) recalled having made a quit attempt lasting at least 24 hours in the previous year, and there was a mean stage of change shift of 0.17.

### GP smoking cessation advice

Among participants who completed follow-up questionnaires at six months, 80/104 (77%) in the intervention group and 68/86 (79%) in the control group had consulted their GP since the baseline consultation (p = 0.7). Recall of smoking cessation advice from their GP during these consultations is summarised by group in Table 5.

**Table 5:** Recall of GP smoking cessation advice in consultations subsequent to the index consultation

	**Intervention group (N = 104) n (%)**	**Control group (N = 86) n (%)**	**p value**
Advised to stop smoking	67 (64)	46 (53)	0.1
Discussed health risks of smoking	57 (55)	48 (56)	0.9
Discussed passive smoking	22 (21)	18 (21)	1.0
Set quit date	12 (12)	7 (8)	0.4
Arranged another appointment to discuss quitting	5 (5)	3 (3)	0.7
Gave practical advice about how to quit smoking	32 (31)	32 (37)	0.3
Offered written information about quitting	41 (39)	34 (40)	1.0
Recommended nicotine replacement gum	17 (16)	12 (14)	0.6
Recommended nicotine replacement patches	27 (26)	22 (26)	1.0
Recommended nicotine replacement inhaler	6 (6)	6 (7)	0.7
Recommended 'Zyban'	12 (12)	10 (12)	1.0
Referred to a smoking cessation clinic or counsellor	5 (5)	5 (6)	0.8

## Discussion

The impact of any intervention is predicated not only upon its efficacy but also its reach and uptake among the target population [39]. Despite designing a smoking cessation intervention that was based on the best available scientific evidence, this randomised trial failed to demonstrate significant improvement in smokers' cessation rates or intention to quit smoking when the intervention was offered opportunistically for patients attending routine consultations in general practice. Among smokers in the intervention group, less than half accepted the offer of referral for telephone-based assistance from a nurse. Only one-third persisted with the intervention.

There is a considerable body of literature that has identified specific smoking cessation strategies that clinicians should use in order to maximise the efficacy of their smoking cessation advice [4,20,22]. Thus, the challenge for tobacco control research is not so much to determine which specific strategies to use, but rather how this knowledge can be implemented to assist the greatest number of smokers in the community. Identification of, and intervention with, smokers attending general practice is as yet an underutilised strategy both in Australia and elsewhere [7-10]. An intervention that capitalises on the reach of general practice to identify smokers but which delegates the time consuming counselling required to another health profession would seem ideal in theory. Regrettably, this was not confirmed. Our results also are consistent with those of the trial undertaken by Ockene and colleagues more than a decade ago in the United States that failed to demonstrate any benefit of telephone counselling as an adjunct to opportunistic physician advice [24].

Previous studies that have shown positive benefits from telephone cessation counselling have predominantly reflected the results that can be achieved with more motivated smokers who actively are seeking such advice through a helpline or who have been deemed eligible to be offered the intervention on the basis of their current intention to quit. In contrast, the aim of the present study was to provide an intervention for any smoker, regardless of their current quit intentions, so as to move all smokers along the stage-of-change continuum towards complete cessation. It was hypothesised that as a number of unsuccessful attempts usually are needed before a person successfully quits, any improvement in intention to quit would signify a measure of success that could improve the chances of successful cessation in the future. While stage of change as measured in this study may not accurately predict future smoking status, it was disappointing that the intervention had no measurable impact on smokers' stated intentions, even in the short term.

One explanation for the lack of demonstrable benefit in this trial was the lower than anticipated acceptance of the intervention by smokers. Only 45% of those offered referral accepted even the first call by the nurse, and only one-third of the intervention group completed all four calls of the intervention. While smokers who were more motivated to quit were significantly more likely to accept and complete the intervention, motivated smokers were no more likely to actually quit than similarly motivated smokers in the control group as the intervention was equally ineffective regardless of patients' baseline stage of change. Among those smokers who completed the intervention, six and twelve month quit rates of 16% and 13% were achieved but the absence of a comparable control group precludes interpretation of these findings as a measure of intervention effectiveness. Future studies could investigate the impact of the intervention among the subgroup of more motivated smokers attending general practice.

Patients in the control group received 'usual care' from their GP. Based on previous research that consistently has demonstrated minimal smoking cessation advice from GPs to smokers during routine consultations [8,9], it is probable that control group patients received, at best, a recommendation to stop smoking and the provision of a 'Quit Kit' to take home. Unfortunately it was not possible to monitor consultations directly as this study was undertaken in non-academic family practices where a requirement to tape consultations would likely have considerably reduced GPs' willingness to take part in the trial. If GPs had inadvertently 'compensated' for patients being allocated to the control group by providing a greater level of advice to these individuals, this could also go some way towards explaining the lack of apparent benefit of the nursing intervention. However, there was no difference between the intervention and control groups in patients' recall of smoking cessation advice in subsequent consultations so it does not appear that GPs differentially provided greater follow up about smoking cessation for those in the control group.

We acknowledge a number of other limitations to this study. Given the lower than expected uptake of the intervention, statistical power was compromised but our data can be used in future meta-analyses. A much larger study is needed to provide a definitive assessment of this intervention. Given the low rates of acceptance of the intervention among smokers however, future studies also ought focus on the identification and provision of intensive support for the subgroup who are motivated to quit. Additional strategies are needed to motivate those smokers who are not yet ready to make a quit attempt. The assumption that all those lost to follow up were continuing smokers is plausible but could have biased the results towards a null finding. We acknowledge considerable debate about the advantages and disadvantages of cotinine validation of self-reported smoking status [28,29]. Yet a requirement for cotinine validation would likely have further increased loss to follow up, with little gain in measurement accuracy.

## Conclusion

This randomised trial failed to demonstrate any impact of opportunistic referral of smokers for telephone-delivered smoking cessation advice by a nurse on subsequent quit rates or smokers' stage of change. Given the lack of evidence to date in support of this approach, it cannot yet be recommended unequivocally.

## Competing interests

The author(s) declare that they have no competing interests.

## Authors' contributions

JY and JW conceived the study and designed the protocol. JY performed the statistical analyses and drafted the manuscript. SG recruited the GPs, managed the study and contributed to data analysis, TB developed the nursing intervention and co-ordinated the data collection, MH provided the nursing intervention. All authors have read and contributed to this article and approved the final manuscript.

## Pre-publication history

The pre-publication history for this paper can be accessed here:


